# The Feasibility of Connecting Conversations: A Narrative Method to Assess Experienced Quality of Care in Nursing Homes from the Resident’s Perspective

**DOI:** 10.3390/ijerph17145118

**Published:** 2020-07-15

**Authors:** Katya Sion, Hilde Verbeek, Erica de Vries, Sandra Zwakhalen, Gaby Odekerken-Schröder, Jos Schols, Jan Hamers

**Affiliations:** 1 Department of Health Services Research, Care and Public Health Research Institute, Maastricht University, Duboisdomein 30, 6229 GT Maastricht, The Netherlands; h.verbeek@maastrichtuniversity.nl (H.V.); erica.devries@maastrichtuniversity.nl (E.d.V.); s.zwakhalen@maastrichtuniversity.nl (S.Z.); jos.schols@maastrichtuniversity.nl (J.S.); jph.hamers@maastrichtuniversity.nl (J.H.); 2 Living Lab in Ageing and Long-Term Care, Duboisdomein 30, 6229 GT Maastricht, The Netherlands; 3 Department of Marketing and Supply Chain Management, School of Business and Economics, Maastricht University, Tongersestraat 53, 6221 LM Maastricht, The Netherlands; g.odekerken@maastrichtuniversity.nl

**Keywords:** narrative, quality assessment, feasibility, interviews, relationship-centered care, quality of care, triad, resident perspective

## Abstract

Currently, residents living in nursing homes and their caring relationships are being placed more centrally in the care experience. Experienced quality of care is influenced by the interactions between residents, family and caregivers, who each have their own experiences and needs. Connecting Conversations is a narrative method aimed at assessing experienced quality of care in nursing homes from the resident’s perspective by having separate conversations with residents, family and caregivers (triads), adopting an appreciative inquiry approach. This study presents how to use Connecting Conversations and its feasibility. Feasibility was assessed as performance completeness, protocol adherence and interviewers’ experiences. Conversations were conducted by trained nursing home staff (n = 35) who performed 275 Connecting Conversations in another nursing home than where they were employed (learning network). Findings show it is feasible to perform separate appreciative conversations with resident–family–caregiver triads by an interviewer employed in another nursing home; however, protocol adherence was sometimes challenging in conversations with residents. Interviewers valued the appreciative approach, the learning network and the depth of the separate conversations. Challenges were experienced with scheduling conversations and receiving time and support to perform the conversations. Stakeholders should continue collaboration to embed Connecting Conversations into daily practice in nursing homes.

## 1. Introduction

The proportion of people over 60 years is expected to almost double from 12% (2015) to 22% (2050) [1]. The aging population has resulted in an increasing number of older people with chronic diseases requiring long-term care [2]. The most vulnerable people with complex health needs live in nursing homes in which they receive 24-h care and functional support [3]. Nursing homes are struggling to maintain and improve their quality of care due to the increase in aging population and strain on resources, the complexity of residents’ needs, the changes in residents’ expectations and the challenges in staff-mix [4,5,6,7]. According to the Institute of Medicine, a component of the US National Academy of Sciences, quality of care needs to be safe, effective, efficient, timely, patient-centered and equitable [8]. It is challenging to fully operationalize these generic concepts to the nursing home setting and therefore quality indicators are often used [9]. To assess these quality indicators, such as the prevalence of pressure ulcers or malnutrition, standardized quantitative methods are used, such as the nursing home minimum data set (MDS) or the national prevalence measurement of quality of care (LPZ) [10,11]. More recently, initiatives such as the Worldwide Elements to Harmonize Research in Long-term Care Living Environments (WE-THRIVE) have occurred, aiming to achieve global common data elements for quality of care to enhance standardized assessments in long-term care [12]. Additionally, specific areas of health care, for example palliative care, have identified their own indicators for quality of care [13]. Stakeholders use quality of care data for different purposes, for example, professional caregivers may use them to learn, reflect and improve care provision, nursing home managers to monitor and improve their performance, and policy makers for transparency and accountability [14,15]. 

In service science, quality is often defined as the comparison of the consumer’s expectations and the actually delivered service, assessed with the outcome ‘satisfaction’ [16]. Care provision in nursing homes can be considered a type of service delivery in which the resident’s expectations and experiences gain a much more important role than in the more traditional quality of care definitions. Evaluations of care services more frequently are trying to fully recognize residents’ needs and experiences with the complete service experience before, during and after receiving care [17]. This means evaluation does not only focus on the actual activity, but also incorporates, for example, how the resident was approached during this activity. By mapping the full customer journey, the sum of all experiences (touchpoints) can be described and moments of truth can be identified that can positively or negatively influence an experience [18]. This holistic view can help care organizations to sustain caring relationships and retention, and receive positive word-of-mouth [17]. 

In line with this service science perspective, residents and their caring relationships are being placed more centrally in the care experience, as can be seen in care models such as person-centered care and relationship-centered care [19,20]. Person-centered care focusses on residents as each being unique human beings with their own needs and wishes, and relationship-centered care goes one step further by focusing on all people involved in the residents’ care experiences, including family, and the impact of their reciprocal relationships [21,22,23]. This concept is known as balanced centricity in service sciences, implying that experiences are created by multiple stakeholders whose needs deserve to be acknowledged [24]. Residents, family and caregivers each have their own experiences and needs and by including all involved stakeholders when assessing quality of care, quality improvement initiatives can focus more on what matters most from a holistic perspective [25,26,27,28]. Additionally, this contributes to a resident’s quality of life and well-being, families feeling valued by making a useful contribution and caregivers’ job satisfaction [29,30]. In line with this holistic view on quality of care, the Dutch policy guidelines for quality of care in nursing homes have been revised to focus more on person- and relationship-centered care, well-being, safety and learning together with and from each other’s practices, highlighting the importance of assessing quality of care from the resident’s perspective [31].

Studies have revealed the complementary value of assessing quality of care by having conversations with residents, their families and professional caregivers, as each have their own needs and stories [25,32]. The addition of the story behind quality rating is often missing when resident experiences and outcomes are only assessed with quantitative patient-reported experience (PREMs), patient-reported outcome (PROMs) and satisfaction measures [33,34,35]. Stories about experiences, so-called narratives, help people to make sense of their world, relationships and themselves, and can support nursing homes to focus on what really matters [35,36]. They can help to identify what is most important to residents and can support quality improvement initiatives for individual residents [37]. Narratives are able to capture an experience that is enriched by incorporating emotions, explaining logic and providing details about the caring relationships [38]. As quality of care is a complex concept, there is a need to assess multiple quantitative and qualitative indicators, and this information should be used in continuous quality improvement cycles [14].

Narratives are already being used as methods to assess for example children’s speech [39] or perform mental health research with young children [40] and in nursing homes as interventions, such as life reviews, to improve residents’ life satisfaction [41,42]. However, the use of narratives as a method to structurally assess elements of quality of care in long-term care is relatively new. This is gradually occurring more frequently; however, little is known about how to use them and their feasibility in practice [43,44]. Recently, the narrative method Connecting Conversations has been developed aimed at assessing experienced quality of care in nursing homes from the resident’s perspective. It was developed according to the steps in the development and evaluation of a measurement method by De Vet, Terwee, Mokkink and Knol [43], including defining the construct to be measured [45], mapping the needs of key stakeholders [46], one cycle of pilot-testing and two cycles of field-testing. This study aimed to present how to use the narrative method ‘Connecting Conversations’ in practice and its feasibility. Validity findings have been published separately in this special issue of IJERPH as well[47].

### Theoretical Foundation

Quality of care from the resident’s perspective, i.e., experienced quality of care, is a process in which expectations occur prior to receiving care, interactions occur during the care experience and an assessment is given after the care experience within a certain context, as defined by the Individually Experienced Quality of Post-Acute and Long-Term Care (INDEXQUAL) framework [45]. Relationship-centered care and caring relationships, individual needs of the resident, family and caregiver (a triad) and their interactions are considered to be at the core of a care experience [22,48,49]. Therefore, to assess experienced quality of care, it is important to ask not only residents, but also family and caregivers how the resident experiences the quality of care, by performing separate conversations [46]. Additionally, the resident’s full customer journey should be considered during quality assessments, as stories, experiences and preferences between residents differ [12,28]. 

It could be beneficial to adopt a positive approach when performing these conversations, as nursing homes often adopt a problem-focused approach magnifying what is not going well; whereas focusing on what is working best and how to build on this can be more rewarding [46,50]. Appreciative inquiry is a positive approach identified as the opposite of problem-solving and helps participants to really engage and focus on discovery (appreciate the best of what is), dream (imagine what could be), design (determine what should be) and destiny (create what will be) [51]. This approach has proven to have positive outcomes on the nursing home culture and interactions by care staff [50,52,53]. The INDEXQUAL framework, relationship-centered care and appreciative inquiry are the theoretical foundation of Connecting Conversations. 

## 2. Materials and Methods 

The study used a cross-sectional design and data collection was performed in two cycles of field-testing: (1) October 2018 to February 2019 and (2) October 2019 to January 2020. First, a description of the content of Connecting Conversations is provided, followed by the operationalization of feasibility, details of the participants, data-collection and data-analysis used to assess feasibility.

### 2.1. Connecting Conversations 

**Figure 1:**
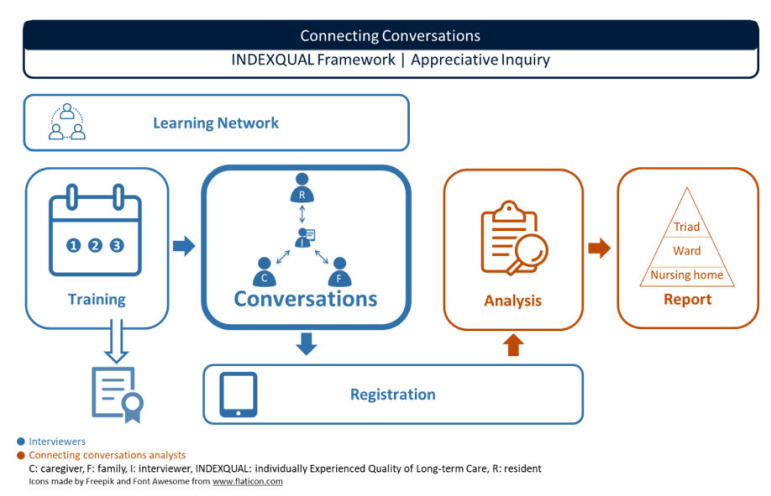
Connecting Conversations.

The narrative method Connecting Conversations aims to assess experienced quality of care in nursing homes from the resident’s perspective. Figure 1 presents the structure of ‘Connecting Conversations’. The content of each blue element is performed by a trained interviewer. The orange elements are currently performed by the research team, as these are still under development. Separate conversations are performed with a resident, family member and professional caregiver of that resident, a so-called care triad. These conversations are registered in an app on a tablet. Interviewers follow a mandatory three-day training to be able to perform the conversations in another nursing home than where they are employed, facilitating a learning network. The research team analyses and reports back the data to the nursing homes. All elements are described in detail in Appendix A. Table 1 provides a brief description of each element.

### 2.2. Interpretation and Operationalization of Feasibility for Connecting Conversations

To determine to what extent it is feasible to use Connecting Conversations in practice, feasibility has been defined as the extent to which Connecting Conversations was conducted as planned and how interviewers experienced Connecting Conversations. This definition has been operationalized into three elements: completeness, protocol adherence and interviewer experiences as presented in Table 2. Feasibility analyses only focused on the Connecting Conversations elements performed by the interviewer: conversations, registration, training and learning network.

### 2.3. Setting and Participants

This study was performed within the Living-Lab in Ageing and Long-Term Care. The living-lab is a collaboration between seven long-term care organizations and four educational institutes, all located in the southern part of the Netherlands [56].

#### 2.3.1. Care Triads

Each of the seven care organizations selected one somatic (for people with physical deterioration) and one psychogeriatric (for people with cognitive decline) ward. Within the selected wards, random selection of residents was necessary to increase the reliability and validity of the assessment and avoid biased selection of only the most well-spoken and satisfied residents with closely involved families. Residents were randomly selected from the nursing home ward by generating a random sequence list of all residents’ room numbers of the selected wards. The contact person of the ward approached residents of the first five (cycle 1) or six (cycle 2) randomly generated room numbers to participate. When a resident refused, the next was approached until the total number of triads was recruited. A family member and professional caregiver closely involved with the selected residents daily care provision were invited, once the resident agreed to participate. Triads were included as dyads if a resident was unable to have the Connecting Conversations because of cognitive impairment (family–professional caregiver dyad) or if no family was available or unwilling to participate (resident-professional caregiver dyad). To provide all residents the opportunity to have a conversation, conversations were attempted with each resident. Only when the resident did not respond at all or merely mumbled answers that could not be understood, the results of the conversation were not included for that triad.

#### 2.3.2. Interviewers

Any interested staff member employed at one of the seven care organizations within the living-lab was invited to apply and each care organization’s management performed final selection. There were three main selection criteria for interviewers: (1) familiar with the nursing home environment, either by providing hands-on care, such as nurses or recreational coaches, or more managerial, such as ward managers or policy makers; (2) good communication skills and natural empathetic abilities; and, (3) involved in or a strong interest in quality assurance. Selection aimed at including two interviewers per care organization per cycle. Additionally, researchers in geriatric nursing science employed at the university, such as health scientists or psychologists, were allowed to participate as well. A minimum of 14 interviewers (two per care organization) and a maximum of 20 interviewers could participate, as this was the maximum attendance to ensure involvement and interaction during the training. The interviewers attended the training and performed the conversations during their working hours, and did not receive any additional incentives.

### 2.4. Data-Collection and Procedure

#### 2.4.1. Connecting Conversations

Appendix A presents the interview guide of questions asked during the separate conversations. Family and professional caregivers were asked to answer the questions, as they believed the resident would. Interviewers were provided a list of probing questions and supportive visuals for the questions asking for a grade to support them during the conversations.

#### 2.4.2. Procedure

The research team assigned interviewers to another care organization than where they were employed, considering travel distance, to enhance the learning network. This prevents confirmation bias, as the interviewer has no prior knowledge of the resident or the performance of the nursing home [57]. Interviewers scheduled five (cycle 1) or three (cycle 2) full triads with a contact person in their assigned care organization. Multiple conversations could be performed a day, estimated at one hour per conversation. Family members could be interviewed by phone, if scheduling a face-to-face conversation was not possible. 

#### 2.4.3. Completeness

For completeness, data from cycle 1 and 2 were collected by documenting the number and duration of performed conversations. Interviewer characteristics were collected at the start of training day 1 with a survey: age in years, sex, job title and years of working experience in the nursing home setting.

#### 2.4.4. Protocol Adherence

Data from cycle 1 were used to assess protocol adherence. The data were collected by audio recording performed conversations with a tablet.

#### 2.4.5. Interviewer Experiences

Interviewers from cycle 1 and 2 were invited to informally evaluate Connecting Conversations at the end of each training day. The trainer asked if interviewers were satisfied with the content, felt engaged, felt confident and if anything should be done differently. After completing all conversations, interviewers were invited to complete a written customer journey about Connecting Conversations, which described all touchpoints that the interviewer experienced during Connecting Conversations in a pre-developed format [18]. The five touchpoints in this journey were (1) the training, (2) scheduling conversations, (3) performing conversations, (4) documenting conversations and (5) miscellaneous for any other comments. Information was gathered adopting an appreciative inquiry approach, asking about what went well during these touchpoints, what could be improved and interviewers’ overall satisfaction. To enhance understanding of what went well and what could be improved, interviewers were invited to attend a group interview or an individual interview, depending on their preference and availability. 

### 2.5. Data-Analysis

#### 2.5.1. Completeness 

Descriptive statistics were used to calculate completeness of all performed conversations, mean duration of conversations and interviewers’ characteristics.

#### 2.5.2. Protocol Adherence

Interviewers’ protocol adherence was evaluated for three elements: (1) the core theme of all six questions was asked; (2) the addressed conversation techniques ‘probing questions’ and ‘paraphrasing’ were applied at least once during each conversation; and, (3) respondents talked more than the interviewer, calculated by the total number of words spoken by the responder divided by the total number of words in the full transcript [58]. These analyses were performed for all conversations of which audio recordings were available (cycle 1). All audio recordings were transcribed verbatim and two researchers scored the transcripts independently. Discrepancies between both researchers regarding if a protocol element was adhered to or not were discussed with a third member of the research team until consensus was reached.

#### 2.5.3. Interviewer Experiences

Interviewers’ evaluations of Connecting Conversations were analyzed and summarized by one researcher with the computer software MAXQDA v20.0.7 [59]. Findings were evaluated with another researcher during two face-to-face discussions. During these discussions, the findings were interpreted and focus was on which elements interviewers appreciated and which were considered challenging. Points for improvement provided during field testing cycle 1 were implemented prior to the start of field-testing cycle 2. The main findings of the evaluations were presented back to the interviewers for validation.

### 2.6. Ethical Considerations

The medical ethics committee of Zuyderland, the Netherlands, approved the study protocol (17-N-86) and concluded that the study was not subject to the Medical Research Involving Human Subjects Act. Information about the study was provided to all interviewers, residents, family members and caregivers in advance by letter. All participants provided written informed consent to contribute to the study and residents with legal representatives gave informed assent themselves before and during the conversations, and their legal representatives gave written informed consent [60]. Participation was strictly voluntarily and participants could withdraw from the study at any moment. Anonymity of participants was guaranteed and therefore no names or organizations were documented, unless participants provided consent to share their individual data with the nursing staff for quality improvement initiatives.

## 3. Results

In total, 35 interviewers attended the training and performed 275 Connecting Conversations (89 residents, 83 family members, 103 caregivers) in 18 different nursing homes (8 psychogeriatric, 9 somatic and 1 acquired brain injury). When residents refused to participate, the most common reason was that they considered this to be too intensive or they were not interested.

### 3.1. Completeness

Random selection of residents’ room numbers was performed successfully in 14 of the 18 nursing homes. The exchange of interviewers between nursing homes, i.e., the learning network, was deemed feasible, as each interviewer performed at least three conversations in their assigned nursing home. Reasons for unsuccessful random selection and challenges with the learning network were organizational challenges in the nursing home. These consisted of a lack of a designated contact person to manage the selection and scheduling of the conversations, a lack of staff and high time pressure, and a lack of understanding of the added value of the conversations and random selection. During cycle 2, the research team made some improvements to the execution of the study compared to cycle 1. They started recruitment earlier and in a more structured manner, with a standardized protocol, a central e-mail address for questions, clearer instructions and timely follow-up to guide the process more thoroughly. Table 3 presents details on the completeness of collected data and interviewer characteristics in total, and separately for field-testing cycles 1 and 2. 

Completeness was 76% of all planned triads/dyads. For 10% (n = 14) of the conversations, the resident was not able to communicate and for 15% (n = 20) of the conversations, family was not willing or available to participate. Additionally, 24% (n = 32) of the triads could not be recruited due to insufficient triads willing to participate on the ward or challenges scheduling conversations with the visiting interviewer. During cycle 2, completeness rates were notably higher than during cycle 1 (84% and 71%, respectively). Median duration of conversations was 17 min.

### 3.2. Protocol Adherence

Table 4 presents the results of the protocol adherence analysis of 125 transcripts performed by 15 interviewers during field-testing cycle 1 (one interviewer had no successful audio recordings).

Results show the questions were asked correctly for 88% of the cases (agreement rate 85%). Compared to the resident group (73%), the completeness of each separate question asked appears higher in the family (92%) and caregiver group (94%). Completeness of all six questions asked was 39% for residents opposed to 74% and 73% for family and caregivers, respectively. Interviewers indicated that in some cases they went off protocol, because the resident had difficulties answering the open-ended questions. When less than four questions were asked correctly, this was because the resident was experiencing difficulties to have a conversation due to cognitive impairment. In almost all conversations, interviewers used at least one probing question (99%) and in a majority of the conversations, paraphrasing was done (69%). In 86% of the conversations, the responder spoke more than the interviewer did; for conversations with family and caregivers, this was almost always (97%–98%).

### 3.3. Interviewer Experiences

Overall, interviewer experiences were very positive; however, they also experienced some challenges. Evaluations were mostly individual interviews (n = 29) and one group interview (n = 6) was performed. First, the valuable aspects interviewers experienced are presented followed by facilitators that can contribute to properly perform assessments with Connecting Conversations.

#### 3.3.1. In-Depth Attention

“Real attention is given to someone”. Interviewers were positive about the conversations, as became apparent from evaluations such as “I really enjoyed doing this” and “the conversations show a valuable overview of someone’s experienced quality of care”. Interviewers were surprised by the in-depth content of the conversations and found it “really special, the stories you hear and the directions they take”. Registration with the app was considered a real asset, interviewers explained, and it was “so easy to use”. Interviewers specifically valued the audio-recordings: “it was nice that audio recordings were made, so I could fully engage in the conversation without feeling the stress of needing to immediately write everything down”.

#### 3.3.2. Narrative Appreciative Inquiry 

“Different from other conversations because of the questions being asked and the positive approach”. Interviewers experienced the benefit of adopting an appreciative approach, as “often, in other conversations, only the negative side is addressed” and “the questions trigger to think positively”. They also appreciated the positive nature of the training and showed this by being actively engaged and enthusiastic. Most were pleasantly surprised by the dynamic set-up of the training and felt they had really learned to perform appreciative conversations. They appreciated how the trainer created a safe environment, the “balance between theory and practice” and how they became “aware of their own listening skills”.

#### 3.3.3. Three Perspectives

“There is a clear difference between perspectives”. Interviewers valued taking the time to have separate conversations with the resident, a family member and a caregiver of that resident and experienced that “the triad gives three different perspectives”. They really encountered the differences and similarities between the perspectives and that it is important to hear each side to a story.

#### 3.3.4. Learning Network

“Valuable to be in another organization”. Interviewers enjoyed having the training together with colleagues from other care organizations and learning from each other. They also enjoyed performing the conversations in another care organization than where they were employed. Some were surprised by the openness of the responders, which was created by the interviewers’ independent status within the nursing home: “I am a stranger to them who comes to interview them, and nevertheless they express themselves and their feelings to quite some extent”. Interviewers also reflected on observations they made whilst visiting the other nursing home. For example, an interviewer shared she saw all caregivers taking their lunchbreak at the same time, leaving residents all alone in the living room. She realized in her ward they also do that, and has now installed an early and a late lunch shift.

#### 3.3.5. Commitment 

“I really enjoyed participating. My manager would really like to embed Connecting Conversations in the whole care organization”. A majority of interviewers has remained engaged with Connecting Conversations after finalizing their conversations. For example, one interviewer had challenging experiences performing conversations as her assigned nursing home faced challenges to schedule conversations on multiple occasions. A follow-up session, however, kept her involved and motivated to stay engaged. Other interviewers have also positively shared their experiences with their managers and quality policy officers, resulting in an increasing demand for Connecting Conversations throughout care organizations.

#### 3.3.6. Scheduling

“It was challenging to reach the contact person and to find suitable days for the conversations, also taking your own work schedule into consideration”. Whereas the valuable aspects of Connecting Conversations are clearly visible, care organizations should be aware that it is a challenging process to implement this new way of assessing quality of care. There was a large variety between interviewers feeling supported or challenged to perform the conversations. This was mainly influenced by the support of one’s own manager and the support of the care organization that was being visited. As interviewers performed conversations elsewhere, they were dependent on a contact person within the visiting care organization who facilitated recruitment of triads and scheduling of conversations. The contact person was considered a crucial element to successfully complete all conversations. 

Based on all feasibility findings, Table 5 presents the facilitators that need to be considered when implementing Connecting Conversations. The elements have been formulated as facilitators, yet when absent, they will be experienced as barriers for successful implementation. First, organizations should adopt a clear vision in which they support this new way of assessing quality of care and provide resources for this. Second, several prerequisites are important to gather rich and valid stories: random selection of triads, external interviewers in the learning network, sufficient time and resources and a contact person on the ward. Last, when performing the conversations, it is important to be as inclusive as possible.

## 4. Discussion

Connecting Conversations assesses experienced quality of care in nursing homes from the resident’s perspective. This article presented how to use the narrative method ‘Connecting Conversations’ and its feasibility. Main findings show it is feasible to perform separate appreciative conversations with a resident, family member and caregiver of that resident by a trained interviewer employed in another nursing home. Protocol adherence was sometimes considered challenging during conversations with residents, as residents did not always seem to understand the questions. Interviewers mostly valued the appreciative approach, the collaboration between care organizations in the learning network and the time they received for in-depth separate conversations with residents, family and caregivers. Challenges were experienced with scheduling the conversations and not all interviewers received the time and support from their care organizations to perform the conversations.

Findings show it is possible to create a learning network in which care organizations exchange staff as interviewers, under the prerequisites that time and support is provided. Whereas it is often said that narratives are considered big time investments [61], our findings show a median duration of only 17 min per conversation and henceforth it is very feasible to perform these conversations. A successful learning network is characterized by sharing knowledge, balancing interests and self-development [62]. This can contribute to the self-development and reflective learning of the interviewers, which henceforth can increase the quality of care in one’s own nursing home [63]. By integrating this appreciative manner of having conversations into the nursing staff’s routines, focus can be shifted from time-based tasks for residents to continuously connecting with residents [61].

Additionally, findings show appreciative inquiry is a useful approach to engage in conversations about quality of care. By adopting an appreciative evaluation of quality of care, a shift is made towards the positive, embracing caregivers to recognize valuable stories and use these positive insights in their future care provision [51]. Appreciative inquiry has successfully been used in other nursing home initiatives too, for example in the implementation of the sensory garden in Norwegian nursing homes [64] or the My Home Life program in the United Kingdom [65,66]. To anchor an appreciative culture, management should reinforce communication and interactions between people, instead of standardized rules and procedures, on all levels of nursing home organizations: strategic, tactic and operational [67]. Leadership could contribute to this, by, for example, assigning Connecting Conversation champions who adopt a key role in successfully developing and supporting quality improvement initiatives based on the collected narrative data [68]. This, in turn, can contribute to increased quality of care and a positive psychosocial climate [69].

Protocol adherence findings confirm the importance of a proper training for interviewers in which they learn how to adhere to the protocol and apply the appreciative approach and conversation techniques. Interviewers’ skills, motivation, reliability, flexibility and productivity contribute in achieving completeness of planned triads [70]. As interviewers are part of a narrative quality assessment method, they play a major role in the reliability of the quality data [71]. Interviewers are not just recorders of the experiences, as they also have an experience of the shared experience [72]. Therefore, to increase the richness of the collected quality of care experiences, it is recommended to invest in proper selection and training of interviewers.

This study shows that a majority of the randomly selected residents living in nursing homes are capable of having a conversation about their experiences. However, complete protocol adherence appeared to be challenging, as in more than half of the conversations, the interviewer was unable to ask all six questions according to protocol. Studies often exclude residents living in nursing homes with a certain degree of dementia or other cognitive declines [73,74,75,76]. It is important to include the resident’s voice and others have confirmed that in most cases, with well-trained interviewers and adapted questions, this is possible [77,78]. For Connecting Conversations, it is recommended to adjust the protocol for residents with cognitive impairment, by for example reformulating the six overarching questions into multiple shorter and easier sub-questions. For an even more inclusive approach, it is recommended to perform additional observations when residents are indeed unable to have the conversation (i.e., very severe dementia or aphasia), to assure their experiences are also fully captured, for example with the Maastricht Electronic Daily Life Observation (MEDLO) tool [32,79]. Other methods that exist for this include Dementia Care Mapping (DCM) or Person. Interaction. Environment. Care Experience in Dementia (PIECE-DEM)[80,81]. The challenges of these observation methods are that they are considered time-consuming and they have not been developed based on the principles of the INDEXQUAL framework of experienced quality of care, but on other theoretical frameworks.

Narratives are considered worth the time investment because they can have a positive impact on the caring relationships between residents, family and their caregivers, and residents’ feelings of autonomy and well-being [61,82]. However, for future implementation, there is room for improvement regarding analysis and reporting of the results. The stories from three perspectives provide rich information that can be used on multiple levels, and the forms of analysis and reporting are dependent on the reason why experienced quality of care is assessed [15,82]. On an operational level, results can provide care teams with directories for continual learning and quality improvements for individual triads and teams. On a tactical level, managers need input on what is going well and what needs improvement within their ward or nursing home. To discover trends on an organization-wide strategic level, other analysis techniques could be more helpful, such as text mining, aimed at analyzing and identifying trends in large amounts of qualitative data [83]. On all these levels, the model of relationship-centered organizations may be a fitting framework to adopt, as it focusses on the web of relationships between care professionals, their actions and cycles of reflection, which is supported by inquiry-centered leadership and a culture of continual learning [84].

Findings show promising results for expanding the use of the narrative assessment method Connecting Conversations in practice. For successful implementation, there are many important determinants that need to be operationalized to the specific intervention and setting, including knowledge and cognition, attitude, routines, social influence, organizational characteristics and resources [85]. Additionally, recent research has shown that developed interventions in the care sector are in need of self-sustaining business models and therefore it is important to develop a suitable business model for Connecting Conversations, keeping its contextual factors into consideration [86]. For high completeness rates, it is important to clearly communicate with the participating interviewers and nursing homes, have clear protocols in place, follow-up in a timely manner and continuously be available to answer questions and provide support.

The current study has not incorporated experiences of how respondents within the triads experienced the new way of assessing quality of care with Connecting Conversations. It is recommended for future research to ask them to describe their experiences with this new way of assessing quality of care from the resident’s perspective, as they are considered the key players in the conversations. Additionally, future research should focus on evaluating Connecting Conversations’ validity and reliability. Further development should combine research with practice and policy to focus on how the information from Connecting Conversations can be reported back to care organizations so the data can be used to improve quality of care in nursing homes. Stakeholders should collaborate to successfully and sustainably embed Connecting Conversations into daily practice in nursing homes.

## 5. Conclusions

To our knowledge, Connecting Conversations is one of the first narrative methods aimed at assessing experienced quality of care in nursing homes as a customer journey, within a triad, from the resident’s perspective in an appreciative way. It would be useful for nursing homes to implement a full quality assessment formula in which clinical and safety indicators, staffs’ job satisfaction and residents’ experienced quality of care are structurally assessed to gain a holistic view on quality of care. This can contribute to providing and receiving the best possible care and working conditions for residents, family and staff.

## Figures and Tables

**Table 1:** A summarized description of the Connecting Conversations elements.

Element	Main Description
Training	Interviewers need to follow a mandatory three-day (3 h/day) training to assure the quality and reliability of performing and registering Connecting Conversations. The training focusses on connecting, practicing and sharing experiences, and has adopted an appreciative inquiry approach. Successful attendance results in a certificate.
Conversations	Semi-structured questions are asked in separate conversation with a resident, family member and professional caregiver of that resident, who each answer from the resident’s perspective. Questions are based on the INDEXQUAL framework and are formulated from an appreciative inquiry approach. Main topics: resident’s life, satisfaction with care provision, most positive experience, description of an average day in the nursing home and relationships between the resident, family and caregiver.
Registration	The Connecting Conversations app supports interviewers to perform, register and view the conversations. Main features app: documenting informed consent, participant demographics, summative answers, audio recording and viewing collected data.
Learning network	The learning network provides a platform for interviewers in which they can learn from and with each other through continuous interaction [54]. Interviewers from different care organizations follow the training together and perform conversations in each other’s care organizations, thus not where they themselves are employed. This provides for independent interviewers and the opportunity for interviewers to learn from daily practices in another nursing home environment.
Analysis	The written texts, as reported in the app, are analyzed by two researchers with content analysis [55].
Report	The analyzed data are presented on ward level in a factsheet with supporting ‘quotes’. Additional reports on triad and nursing home level can be delivered upon request.

**Table 2:** Feasibility definitions, operationalization and analyses for Connecting Conversations.

Feasibility Concept	Definition	Operationalization for Connecting Conversations Element Analyzed	Analysis
Completeness	Extent to which Connecting Conversations was completed as planned	All planned triads were randomly selected and completed in the learning network as planned Interviewers completed the training and all planned conversations Conversations Learning network	Description of successes and challenges of random selection of triads on a ward and the learning network Completed conversations rate ^1^, including documentation of incomplete and missing triads, and the duration of the conversations Description of recruited interviewers and attendance rate ^1^ training
Protocol adherence	Extent to which the conversations were performed as planned	All interviewers followed the Connecting Conversations’ protocol as taught during the training. Conversations Training	All six questions were asked as formulated in the protocol ^1^ Per conversation at least one probing question and one time paraphrasing was used ^1^ The respondent talked more than the interviewer ^1^
Interviewer experiences	Interviewers’ satisfaction with Connecting Conversations and experienced facilitators and barriers	All interviewers evaluated all components of Connecting Conversations: training, scheduling conversations, performing conversations and registering conversations. Conversations Registration Training Learning network	Deductive coding of interviewer experiences, categorized into elements that were appreciated and that were considered challenging

^1^ Interpret as total percentage of participants: <60% not acceptable, 60%–80% acceptable, >80% good.

**Table 3:** Connecting Conversations’ care triads and interviewer demographics.

Care Triads	Total	Field-Testing Cycle 1	Field-Testing Cycle 2
Planned conversations n			
→ Total	405	240	165
→ Triads R-F-C	135	80	55
Performed conversations n (%)			
→ Total	275 (68) ^3^	149 (62) ^5^	126 (76) ^7^
→ Resident (R)	89 (66)	46 (58)	43 (78)
→ Family (F)	83 (61)	46 (58)	37 (67)
→ Caregiver (C)	103 (76)	57 (71)	46 (84)
→ Total triads/dyads	103 (76)	57 (71)	46 (84)
→ Full triads R-F-C	68 (50) ^4^	34 (43) ^6^	34 (60) ^8^
→ F-C combination ^1^	14 (10)	11 (14)	3 (5)
→ R-C combination	20 (15)	11 (14)	9 (16)
→ Full triads missing	32 (24)	23 (29)	9 (16)
Mean/Median minutes conversations (range)			
→ Total	19/17 (3–79)	18/15 (3–54)	21/18 (4–79)
→ Resident (R)	21/17 (4–79)	18/14 (6–54)	24/22 (4–79)
→ Family (F)	21/19 (6–48)	21/22 (6–39)	21/18 (7–48)
→ Caregiver (C)	17/14 (3–55)	15/14 (3–41)	19/16 (4–55)
**Interviewers’ characteristics**			
Total interviewers n	35	16	19
Mean age in years (SD)	40 (11)	40 (11)	42 (11)
Females (%)	31 (89)	14 (88)	17 (89)
Occupation n (%)			
→ Nurse	10 (29)	6 (38)	4 (21)
→ Baccalaureate-educated nurse	9 (26)	4 (25)	5 (26)
→ Policy advisor	5 (14)	3 (19)	2 (11)
→ Care manager	2 (6)	0	2 (11)
→ Recreational coach	2 (6)	0	2 (11)
→ Psychologist ^2^	3 (9)	1 (6)	2 (11)
→ Health scientist ^2^	2 (6)	1 (6)	1 (5)
→ Nurse aid	1 (3)	1 (6)	0
→ Complaints officer	1 (3)	0	1 (5)
Mean contracted hours per week (SD)	32.4 (5.2)	32.3 (5.2)	32.6 (5.3)
Mean years working experience (SD)	13.1 (11.0)	13.8 (9.7)	12.4 (12.1)
Training attendance all 3 days n (%)	30 (86)	13 (81)	17 (89)
Training attendance 2 out of 3 days n (%)	5 (14)	3 (19)	2 (11)

^1^ Residents missing because on psychogeriatric ward and not cognitively capable to have the conversation. ^2^ Not employed at the nursing home, but at the university. ^3^ Of which 241 with audio recordings. ^4^ Of which 52 with audio recordings. ^5^ Of which 125 with audio recordings. ^6^ Of which 24 with audio recordings. ^7^ Of which 116 with audio recordings. ^8^ Of which 28 with audio recordings.

**Table 4:** Protocol adherence results ^1^.

	Total	Resident (R)	Family (F)	Caregiver (C)
	N = 125	N = 36	N = 38	N = 51
Question 1 quality of life n (%)	107 (86)	24 (67)	36 (95)	47 (92)
Question 2 satisfaction caregivers n (%)	113 (90)	29 (81)	34 (89)	50 (98)
Question 3 most positive n (%)	116 (93)	30 (83)	36 (95)	50 (98)
Question 4 average day n (%)	113 (90)	26 (72)	37 (97)	50 (98)
Question 5 relationships n (%) ^2^	102 (82)	24 (67)	34 (89)	44 (86)
Question 6 relationships n (%) ^3^	106 (85)	25 (69)	33 (87)	48 (94)
Average questions asked %	88	73	92	94
All six questions asked n (%)	79 (63)	14 (39)	28 (74)	37 (73)
Four or five questions asked n (%)	30 (24)	10 (28)	8 (21)	14 (27)
Less than four questions asked n (%)	14 (11)	12 (33)	2 (5)^4^	0
Probing questions n (%)	124 (99)	36 (100)	37 (97)	51 (100)
Paraphrasing n (%)	86 (69)	22 (61)	29 (76)	35 (69)
≥50% responder words spoken n (%)	108 (86)	23 (64)	37 (97)	50 (98)

^1^ Interpret as total percentage of participants: <60% not acceptable, 60-80% acceptable, >80% good. ^2^ Relationships: resident (resident–caregiver), family (family–caregiver), caregiver (caregiver–resident).^3^ Relationships: resident (resident–family), family (family–resident), caregiver (caregiver–family). ^4^ This interview was performed by one interviewer that did not adhere to protocol.

**Table 5:** Facilitators to implement Connecting Conversations.

	Facilitators	Reason Why Important
Vision	Adopt an appreciative inquiry approach when introducing, implementing and embedding Connecting Conversations into the nursing home	To enhance commitment and enthusiasm; and set an example of the method’s positive impact: ‘practice what you preach’
	Have a clear purpose for what the results will be used	To decide on the magnitude of the assessment and the format of the report(s)
Prerequisites	Random selection of triads on a ward	To avoid selection bias
	Assure interviewers have conversations elsewhere than where they are employed (external interviewers)	To enhance the learning network and provide respondents a safe environment to share their stories
	Provide sufficient time for training, conversations and the learning network	To ensure quality of the conducted conversations
	Assign a contact person in the nursing home who is responsible for facilitating the visiting interviewer (scheduling conversations; informing residents, family and staff on the ward)	To enhance completeness and to create a safe environment for the visiting interviewer
Performance	Make an effort to have conversations with each selected resident, regardless of his or her (cognitive) health status	To embrace an inclusive approach, in which residents are provided with self-determination
	Think in solutions when scheduling conversations, for example by allowing full-time employed family to have the conversation by phone or during evening hours	To embrace an inclusive and appreciative approach

**Figure A1:**
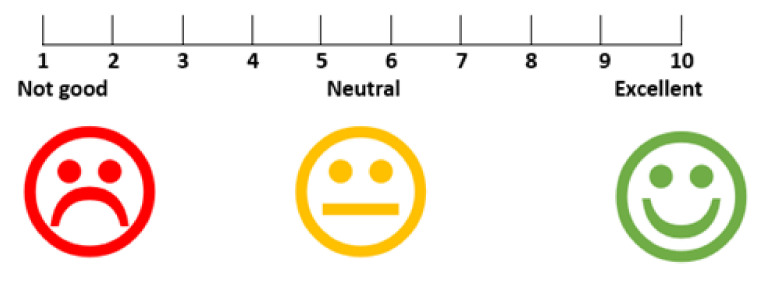
Supportive visual for Connecting Conversations.

**Figure A2:**
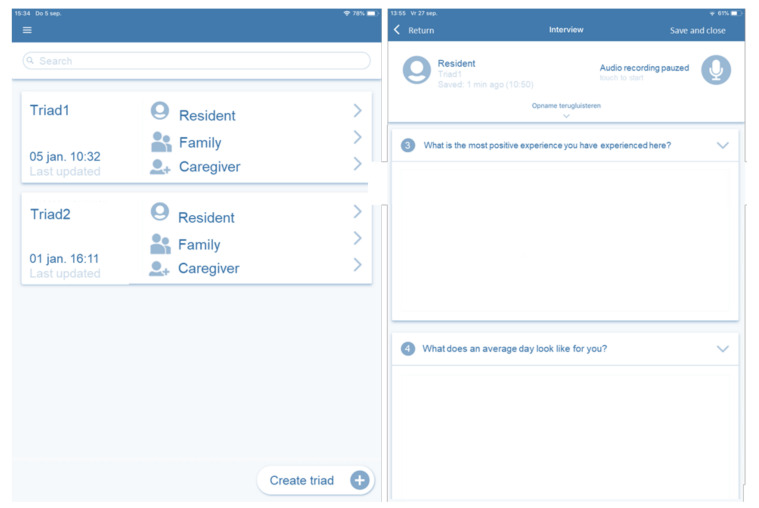
Screen shots from the Connecting Conversations app: triad list (left) and conversation with resident (right).

## Appendix A

This appendix presents a full description of Connecting Conversations, as briefly presented in Figure 1 and Table 1. Connecting Conversations aims to assess experienced quality of care in nursing homes from the resident’s perspective.

### Appendix A.1. Conversations

Table A1 presents the semi-structured questions that are asked during Connecting Conversations, providing interviewers guidance throughout the conversations. Family and professional caregivers are asked to answer the questions, as they believe the resident would. Questions 1 to 4 replace “you” with “your loved one” for family and “resident’s name” for caregivers. Questions 5 and 6 are adapted to reflect the respondents’ relationships, thus family are asked about their contact with the resident and the caregivers; and caregivers are asked about their contact with the resident and the family.

**Table A1:** Connecting Conversations’ Questions.

1a 1b	On a scale of 1 to 10, how would you grade your life at this moment? What is needed to make that a [grade +1]?
2a 2b	On a scale of 1 to 10, how would you grade the caregivers that are involved with your daily care provision? What is needed to make that a [grade +1]?
3	What is the most positive experience you have experienced here?
4	What does an average day look like for you?
5a 5b	What is pleasant about your contact with the caregivers here? What could be different about your contact with the caregivers here?
6a 6b	What is pleasant about your contact with your family? What could be different about your contact with the family here?
7a 7b	What goes well here? What could be done more here?
8	Is there anything left you would like to share that has not been addressed yet?
Probing questions	Why? What is going well? What could be done more? How did that make you feel? Can you give an example?

All questions are based on the elements of the INDEXQUAL framework, capture the resident’s customer journey and are formulated from an appreciative inquiry approach. The critical incidence technique is applied in question 3 by asking explicitly about the most positive experience, aimed at identifying a critical incident [87]. A critical incident combines cognitive, affective and behavioral dimensions by describing the experience itself, the behaviors of everyone involved and the result of these behaviors [88]. Question 4 provides respondents the opportunity to fabricate their own customer journey, which contributes to understanding what is important to the resident, family and/or caregiver [18]. Interviewers are provided with a list of probing questions, to support them during the conversations and supportive visuals for the questions asking for a grade (Figure A1).

#### Care Triads Recruitment

On a ward consisting of 15–30 residents, six residents with their family and caregivers are randomly selected to participate by the research team. Care organizations are free to select the nursing home ward, however the research team randomly selects the six residents on the ward, to avoid selection bias. A random sequence list of all residents’ room numbers of the selected wards is generated. When a resident refuses to participate, the next is approached until the total number of triads is recruited. A closely involved family member and professional caregiver are invited to participate, once the resident has agreed.

### Appendix A.2. Registration

Connecting Conversations includes an app for tablets and computers. This app supports interviewers to perform, register and view their Connecting Conversations. The main features of the app are:

- signing informed consent;
- collecting participant demographics;
- presenting semi-structured questions and suggestions for probing questions;
- typing summative answers to each question;
- audio recording and replaying of conversations;
- viewing collected data through a web portal.

Replaying of audio and typing the summative answers can also be done on a computer or laptop by the interviewer, after having performed the conversation. On an online portal managed by the research team, new interviewers and nursing homes can be assigned and the data is securely stored. The raw data as entered into the app are also available for nursing homes upon request, if participants have provided consent for this as it may breach anonymity. Each interviewer has an own secured account in which triads can be created. The app is available in the app Store for tablets and interviewers receive login details during the first training day. Figure A2 presents two screen shots of the app: left shows the list of created triads and right shows the questions, answer fields and audio recording option for a conversation with a resident.

### Appendix A.3. Training

In order to successfully perform and register Connecting Conversations, interviewers need to follow a mandatory three-day training. It aims to assure the quality and reliability of the conversations regardless which interviewer performs a conversation. The training teaches interviewers how to perform Connecting Conversations, focusing on both the theoretical foundations of INDEXQUAL, relationship-centered care, appreciative inquiry and the customer journey, and the practical aspects, such as how to use the app. The training consists of three 3-h sessions in a group of maximally 20 interviewers. Session 1 (day 1) is focused on engaging the group of interviewers, session 2 (day 8) on practicing conversations and session 3 (day 35) on evaluating and reflecting on each other’s first experiences with the conversations. Interviewers are taught how to perform appreciative conversations with residents, family and caregivers, and how to ask probing questions, paraphrase and really listen without making assumptions.

The training is provided by an external company experienced in developing and providing innovative, scientific, tailor-made trainings, adopting an appreciative inquiry approach (in the Netherlands we collaborated with UMIO, an executive branch of Maastricht University). A holistic approach has been adopted, by applying the integral theory of consciousness focusing on intentional (I), behavioral (IT), cultural (WE) and social (ITS) quadrants [89]. The training aims to tackle all four components, to achieve successful long-term change. Whereas standard trainings are often aimed at ‘predict and control’, this training uses a ‘sense and respond’ approach, providing the group space to adjust the content of the training to their personal needs, which enhances engagement and effective use of time [90].

### Appendix A.4. Certificate

Interviewers are rewarded with a certificate if they attend all three sessions and perform at least one triad in another nursing home than where they are employed. Interviewers, who are unable to attend one of the training sessions, receive the opportunity to hand in a compensation assignment. The certificate is valid for 1 years and can be extended after attending a celebration session. A celebration session is organized after all interviewers finalize their interviewers, to share experiences, enhance enthusiasm and future commitment, embrace the learning network, share feedback to further improve, and support interviewers to become Connecting Conversations champions within their organizations.

### Appendix A.5. Learning Network

The learning network aims at contributing to sustainable success by providing a platform for interviewers in which they can learn from each other through continuous interaction [54]. Interviewers from different care organizations follow the training together and perform conversations in each other’s care organizations, thus not where they themselves are employed. This provides them the opportunity to interact with and learn from each other. Additionally, it supports responders in the triads to answer honestly, as the interviewer is independent and not related to the care organization.

### Appendix A.6. Analysis

The written texts as reported in the App, are analyzed by two researchers with content analysis [55]. The texts are formatted in a table consisting of four columns allowing for comparison of answers within an individual triad (Table A2):

(1) the questions asked;
(2) summative answer resident;
(3) summative answer family;
(4) summative answer caregiver.

**Table A2:** Example answer output Connecting Conversations.

Q2. On a scale of 1 to 10, how would you grade the caregivers that are involved with your daily care provision?	*“9, because they do everything they can. It’s just those girls have little time. But they need to see residents within a certain time and cannot just sit around with you.”*	*“Insufficient, because in her opinion very many care providers do not treat her as a person, but as a thing that needs to be dressed quickly.”*	*“8, because the wishes of the client are met, for example breakfast in bed and care is provided later.”*

First, researchers code meaningful segments per triad and label these as ‘this is going well’ (discover) or ‘this needs to be done more frequently’ (dream), adopting an appreciative inquiry approach. Second, they check to what extent the resident, family and caregiver expressed similar or different thoughts within a triad (relationship-centered care). Last, similarities and differences between triads are compared and aggregated into trends that are recognized as going well and that could be done more frequently on a ward, resulting in a report for the nursing home. Both researchers discuss their findings and conflicts with a third member of the research team. It is deemed unsustainable to analyze full transcripts for these large amounts of data, as this is very time-consuming and nursing homes want quick quality improvement cycles.

### Appendix A.7. Report

The research team is responsible for reporting results back to the nursing homes. The analyzed data are presented on ward level in a factsheet with supporting ‘quotes’ by a researcher on location. Nursing homes can choose who attends this presentation, for example the ward manager, nursing home manager, quality policy officer of the nursing home and/or the care team. The presentation consists of eight sections presented from an appreciative inquiry approach and tailored to each ward’s results presented in Table A3.

**Table A3:** Outline of report.

1	Core Principles of Connecting Conversations
**2**	Details on how many conversations were performed in which ward
**3**	To what degree were there many similarities or differences between the resident, family and caregiver within each triad?
**4**	What is going well on the ward? (discover)
**5**	Quotes supporting results on Section 4
**6**	What could be done more frequently on the ward? (dream)
**7**	Quotes supporting results on Section 5
**8**	Discussion asking attendees what they think of the results, what they can learn from the results and what they are going to do with the results?

The ward manager is advised to share the results with the care team, family and residents; and to discuss if the results are familiar, how the team can learn from these results and what actions can be taken based on the findings (design and destiny). On request, nursing homes can ask for additional reports, such as a poster with the main results to share on the ward or a written report that can be used for accountability purposes.

## References

[1] World Health Organisation Ageing and Health: Key Facts. https://www.who.int/news-room/fact-sheets/detail/ageing-and-health.

[2] World Health Organisation (2015). World Report on Ageing and Health.

[3] Sanford A.M., Orrell M., Tolson D., Abbatecola A.M., Arai H., Bauer J.M., Cruz-Jentoft A.J., Dong B., Ga H., Goel A. (2015). An international definition for “nursing home”. J. Am. Med. Dir. Assoc..

[4] OECD/EU (2013). A Good Life in Old Age?.

[5] Miller S.C., Miller E.A., Jung H.Y., Sterns S., Clark M., Mor V. (2010). Nursing home organizational change: The “Culture Change” movement as viewed by long-term care specialists. Med. Care Res. Rev. MCRR.

[6] Zimmerman S., Shier V., Saliba D. (2014). Transforming nursing home culture: Evidence for practice and policy. Gerontologist.

[7] Nakrem S., Vinsnes A.G., Seim A. (2011). Residents’ experiences of interpersonal factors in nursing home care: A qualitative study. Int. J. Nurs. Stud..

[8] Institute of Medicine Committee on Quality of Health Care in America (2001). Crossing the Quality Chasm: A New Health System for the 21st Century.

[9] Castle N., Ferguson J. (2010). What is nursing home quality and how is it measured?. Gerontologist.

[10] Van Nie-Visser N.C., Schols J.M., Meesterberends E., Lohrmann C., Meijers J.M., Halfens R.J. (2013). An international prevalence measurement of care problems: Study protocol. J. Adv. Nurs..

[11] Rahman A.N., Applebaum R.A. (2009). The Nursing Home Minimum Data Set Assessment Instrument: Manifest Functions and Unintended Consequences—Past, Present, and Future. Gerontologist.

[12] Edvardsson D., Baxter R., Corneliusson L., Anderson R.A., Beeber A., Boas P.V., Corazzini K., Gordon A.L., Hanratty B., Jacinto A. (2019). Advancing Long-Term Care Science Through Using Common Data Elements: Candidate Measures for Care Outcomes of Personhood, Well-Being, and Quality of Life. Gerontol. Geriatr. Med..

[13] De Roo M.L., Leemans K., Claessen S.J.J., Cohen J., Pasman H.R.W., Deliens L., Francke A.L. (2013). Quality Indicators for Palliative Care: Update of a Systematic Review. J. Pain Symptom Manag..

[14] Mor V., Leone T., Maresso A. (2014). Regulating Long-Term Care Quality: An. International Comparison.

[15] Clarke A., Rao M. (2004). Developing quality indicators to assess quality of care. Qual. Saf. Health Care.

[16] Lewis R.C., Booms B.H. (1983). The marketing aspects of service quality. Emerg. Perspect. Serv. Mark..

[17] Voorhees C.M., Fombelle P.W., Gregoire Y., Bone S., Gustafsson A., Sousa R., Walkowiak T. (2017). Service encounters, experiences and the customer journey: Defining the field and a call to expand our lens. J. Bus. Res..

[18] Lemon K.N., Verhoef P.C. (2016). Understanding Customer Experience Throughout the Customer Journey. J. Mark..

[19] McCormack B., Roberts T., Meyer J., Morgan D., Boscart V. (2012). Appreciating the ‘person’ in long-term care. Int. J. Older People Nurs..

[20] Wilberforce M., Challis D., Davies L., Kelly M.P., Roberts C., Clarkson P. (2017). Person-centredness in the community care of older people: A literature-based concept synthesis. Int. J. Soc. Welf..

[21] Koren M.J. (2010). Person-centered care for nursing home residents: The culture-change movement. Health Aff..

[22] Duffy J.R., Hoskins L.M. (2003). The Quality-Caring Model: Blending dual paradigms. ANS Adv. Nurs. Sci..

[23] Beach M.C., Inui T. (2006). Relationship-centered care. A constructive reframing. J. Gen. Intern. Med..

[24] Gummesson E. (2008). Extending the service-dominant logic: From customer centricity to balanced centricity. J. Acad. Mark. Sci..

[25] Nolan M., Brown J., Davies S., Nolan J., Keady J. (2006). The Senses Framework: Improving Care for Older People through A Relationship-Centred Approach.

[26] OECD (2017). Ministerial Statement: The Next Generation of Health Reforms.

[27] Nadash P., Hefele J., Wang J., Barooah A. (2017). Nursing home satisfaction measures: What is their relationship to quality?. Innov. Aging.

[28] Corazzini K.N., Anderson R.A., Bowers B.J., Chu C.H., Edvardsson D., Fagertun A., Gordon A.L., Leung A.Y.M., McGilton K.S., Meyer J.E. (2019). Toward Common Data Elements for International Research in Long-term Care Homes: Advancing Person-Centered Care. J. Am. Med. Dir. Assoc..

[29] Kellett U. (1999). Searching for new possibilities to care: A qualitative analysis of family caring involvement in nursing homes. Nurs. Inq..

[30] McGilton K.S., Boscart V.M. (2007). Close care provider-resident relationships in long-term care environments. J. Clin. Nurs..

[31] Zorginstituut Nederland (2017). Kwaliteitskader Verpleeghuiszorg Samen Leren en Verbeteren.

[32] Curyto K.J., Van Haitsma K., Vriesman D.K. (2008). Direct observation of behavior: A review of current measures for use with older adults with dementia. Res. Gerontol. Nurs..

[33] Weldring T., Smith S.M.S. (2013). Patient-Reported Outcomes (PROs) and Patient-Reported Outcome Measures (PROMs). Health Serv. Insights.

[34] Zuidgeest M., Delnoij D.M.J., Luijkx K.G., de Boer D., Westert G.P. (2012). Patients’ experiences of the quality of long-term care among the elderly: Comparing scores over time. BMC Health Serv. Res..

[35] LaVela S.L., Gallan A.S. (2014). Evaluation and measurement of patient experience. Patient Exp. J..

[36] Kenyon G., Randall W. (2015). Introduction. J. Aging Stud..

[37] Heliker D.M. (1997). A Narrative Approach to Quality Care in Long-Term Care Facilities. J. Holist. Nurs..

[38] Finucane M.L., Martino S.C., Parker A.M., Schlesinger M., Grob R., Cerully J.L., Rybowski L., Shaller D. (2018). A framework for conceptualizing how narratives from health-care consumers might improve or impede the use of information about provider quality. Patient Exp. J..

[39] Beswick N. (2008). Determination of the inter-rater reliability of the Edmonton Narrative Norms Instrument. Department of Speech Pathology and Audiology.

[40] Bettmann J.E., Lundahl B.W. (2007). Tell me a story: A review of narrative assessments for preschoolers. Child. Adolesc. Soc. Work J..

[41] Hendriks L., Veerbeek M.A., Volker D., Veenendaal L., Willemse B.M. (2019). Life review therapy for older adults with depressive symptoms in general practice: Results of a pilot evaluation. Int. Psychogeriatr..

[42] Butler R.N. (1963). The Life Review: An Interpretation of Reminiscence in the Aged. Psychiatry.

[43] De Vet H.C.W., Terwee C.B., Mokkink L.B., Knol D.L. (2011). Measurement in Medicine: A Practical Guide.

[44] Triemstra M.F.A. (2017). Literatuurstudie en Overzicht van Instrumenten Kwaliteit van Leven en Zorg Meten.

[45] Sion K.Y.J., Haex R., Verbeek H., Zwakhalen S.M.G., Odekerken-Schröder G., Schols J.M.G.A., Hamers J.P.H. (2019). Experienced Quality of Post-Acute and Long-Term Care From the Care Recipient’s Perspective—A Conceptual Framework. J. Am. Med. Dir. Assoc..

[46] Sion K.Y.J., Verbeek H., de Boer B., Zwakhalen S.M.G., Odekerken-Schröder G., Schols J.M.G.A., Hamers J.P.H. (2020). How to assess experienced quality of care in nursing homes from the client’s perspective: Results of a qualitative study. BMC Geriatr..

[47] Sion K.Y.J., Verbeek H., Aarts S., Zwakhalen S.M.G., Odekerken-Schröder G., Schols J.M.G.A., Hamers J.P.H. (2020). The Validity of Connecting Conversations: A Narrative Method to Assess Experienced Quality of Care in Nursing Homes from the Resident’s Perspective. Int. J. Environ. Res. Public Health.

[48] Soklaridis S., Ravitz P., Nevo G.A., Lieff S. (2016). Relationship-centred care in health: A 20-year scoping review. Patient Exp. J..

[49] Nolan M.R., Davies S., Brown J., Keady J., Nolan J. (2004). Beyond person-centred care: A new vision for gerontological nursing. J. Clin. Nurs..

[50] Cooperrider D., Srivastva S. (1987). Appreciative Inquiry in Organizational Life. Res. Organ. Chang. Dev..

[51] Cooperrider D.L., Whitney D.K., Stavros J.M. (2003). Appreciative Inquiry Handbook.

[52] Dewar B., MacBride T. (2017). Developing caring conversations in care homes: An appreciative inquiry. Health Soc. Care Community.

[53] Beauchamp J.M., Glessner T.M. (2006). Appreciative Inquiry Promotes Nursing Culture Change. Clin. Nurse Spec..

[54] Wenger E. (1998). Communities of Practice: Learning, Meaning, and Identity.

[55] Hsieh H.F., Shannon S.E. (2005). Three approaches to qualitative content analysis. Qual. Health Res..

[56] Verbeek H., Zwakhalen S.M.G., Schols J.M.G.A., Kempen G.I.J.M., Hamers J.P.H. (2019). The Living Lab in Ageing and Long-Term Care: A Sustainable Model for Translational Research Improving Quality of Life, Quality of Care and Quality of Work. J. Nutr. Health Aging.

[57] Nickerson R.S. (1998). Confirmation Bias: A Ubiquitous Phenomenon in Many Guises. Rev. Gen. Psychol..

[58] DeMarrais K.B., Lapan S.D. (2004). Qualitative Interview Studies: Learning Through Experience. Foundations for Research: Methods of Inquiry in Education and the Social Sciences.

[59] MAXQDA (1989–2020). Software for Qualitative Data Analysis.

[60] Black B.S., Rabins P.V., Sugarman J., Karlawish J.H. (2010). Seeking assent and respecting dissent in dementia research. Am. J. Geriatr. Psychiatry Off. J. Am. Assoc. Geriatr. Psychiatry.

[61] Villar F., Serrat R. (2017). Changing the culture of long-term care through narrative care: Individual, interpersonal, and institutional dimensions. J. Aging Stud..

[62] Meerveld E.V., Vos F.S.M., Bos E.H., Jansen Y.J.F.M. (2014). Meerwaarde van een Lerend Netwerk, Casus National Inzetbaarheidsplan.

[63] Boyd E.M., Fales A.W. (1983). Reflective Learning: Key to Learning from Experience. J. Humanist. Psychol..

[64] Magnussen I.-L., Alteren J., Bondas T. (2019). Appreciative inquiry in a Norwegian nursing home: A unifying and maturing process to forward new knowledge and new practice. Int. J. Qual. Stud. Health Well Being.

[65] NCHR&D (2007). Quality of Life in Care Homes: A Review of the Literature.

[66] Dewar B., Nolan M. (2013). Caring about caring: Developing a model to implement compassionate relationship centred care in an older people care setting. Int. J. Nurs. Stud..

[67] Anderson R.A., Issel L.M., McDaniel R.R. (2003). Nursing homes as complex adaptive systems: Relationship between management practice and resident outcomes. Nurs. Res..

[68] Woo K., Milworm G., Dowding D. (2017). Characteristics of quality improvement champions in nursing homes: A systematic review with implications for evidence-based practice. Worldviews Evid. Based Nurs..

[69] Backman A., Sjögren K., Lindkvist M., Lövheim H., Edvardsson D. (2016). Towards person-centredness in aged care—Exploring the impact of leadership. J. Nurs. Manag..

[70] Stiegler A., Biedinger N. (2016). Interviewer Skills and Training. GESIS Surv. Guidel..

[71] Merriam S.B. (1998). Qualitative Research and Case Study Applications in Education. Revised and Expanded from “Case Study Research in Education.

[72] Clandinin D.J., Connelly F.M. (2000). Narrative Inquiry: Experience and Story in Qualitative Research.

[73] Chang S.J. (2013). Lived Experiences of Nursing Home Residents in Korea. Asian Nurs. Res..

[74] Chuang Y.H., Abbey J.A., Yeh Y.C., Tseng I.J., Liu M.F. (2015). As they see it: A qualitative study of how older residents in nursing homes perceive their care needs. Collegian.

[75] Drageset J., Haugan G., Tranvag O. (2017). Crucial aspects promoting meaning and purpose in life: Perceptions of nursing home residents. BMC Geriatr..

[76] Walker H., Paliadelis P. (2016). Older peoples’ experiences of living in a residential aged care facility in Australia. Australas. J. Ageing.

[77] Applebaum R., Uman C., Straker J. (2006). Capturing the voices of consumers in long-term care: If you ask them they will tell. Consumer Voice and Choice in Long-Term Care.

[78] Milte R., Huynh E., Ratcliffe J. (2019). Assessing quality of care in nursing homes using discrete choice experiments: How does the level of cognitive functioning impact upon older people’s preferences?. Soc. Sci. Med..

[79] De Boer B., Beerens H.C., Zwakhalen S.M., Tan F.E., Hamers J.P., Verbeek H. (2016). Daily lives of residents with dementia in nursing homes: Development of the Maastricht electronic daily life observation tool. Int. Psychogeriatr..

[80] Brooker D. (2010). Dementia Care Mapping. Principles and Practice of Geriatric Psychiatry.

[81] Brooker D., La Fontaine J., De Vries K., Latham I. (2013). The development of PIECE-dem: Focussing on the experience of care for people living with advanced dementia. Br. Psychol. Soc. Clin. Psychol. Forum.

[82] Bohlmeijer E., Kenyon G., Randall W. (2011). Toward a Narrative Turn in Health Care. Storying Later Life: Issues, Investigations, and Interventions in Narrative Gerontology.

[83] Usai A., Pironti M., Mital M., Mejri C.A. (2018). Knowledge discovery out of text data: A systematic review via text mining. J. Knowl. Manag..

[84] Safran D.G., Miller W., Beckman H. (2006). Organizational dimensions of relationship-centered care theory, evidence, and practice. J. Gen. Intern. Med..

[85] Van Achterberg T., Schoonhoven L., Grol R. (2008). Nursing implementation science: How evidence-based nursing requires evidence-based implementation. J. Nurs. Scholarsh. Off. Publ. Sigma Tau Int. Honor Soc. Nurs..

[86] Christie H.L., Martin J.L., Connor J., Tange H.J., Verhey F.R.J., de Vugt M.E., Orrell M. (2019). eHealth interventions to support caregivers of people with dementia may be proven effective, but are they implementation-ready?. Internet Interv..

[87] Flanagan J.C. (1954). The critical incident technique. Psychol. Bull..

[88] Serrat O. (2017). The Critical Incident Technique. Knowledge Solutions: Tools, Methods, and Approaches to Drive Organizational Performance.

[89] Wilber K. (1997). An integral theory of consciousness. J. Conscious. Stud..

[90] Bradley S.P., Nolan R.L. (1998). Sense and Respond: Capturing Value in the Network Era.

